# Decade-Stratified Malignancy Risk in Hysteroscopic Polypectomy: A Multicenter Registry Analysis of 9269 Procedures

**DOI:** 10.3390/cancers18193206

**Published:** 2026-10-04

**Authors:** Rina Tamir Yaniv, Tamar Tzur, Hila Goldstein, Gadi Ben-Shitrit, Gabi Haran

**Affiliations:** 1 Department of Gynecology, Assuta Medical Centers, Tel Aviv 6971028, Israeldr.harangabi@gmail.com (G.H.); 2 Faculty of Medical and Health Sciences, Tel Aviv University, Tel Aviv 6997801, Israel

**Keywords:** endometrial polyp, polypectomy, malignancy risk, age, postmenopausal bleeding, indication-stratified, obesity

## Abstract

Background: Malignancy risk in hysteroscopic polypectomy specimens rises with age and postmenopausal status, but decade-stratified absolute rates from large, unselected cohorts remain scarce, limiting the quantitative basis for pre-procedural risk counseling. Objective: To estimate decade-specific absolute malignancy rates and to determine how the documented procedural indication modifies that risk. Methods: Retrospective multicenter cohort study with SNOMED-coded histology. All hysteroscopic polypectomies at five Assuta Medical Center sites from January 2019 to April 2026 with a linked histology result constituted the analytic cohort (*n* = 9269; 75.1% of a base cohort of 12,339). The primary outcome was histologically confirmed endometrial malignancy, defined by SNOMED morphology and topography coding. Multivariable logistic regression adjusted for weight category, hospital site, and calendar year. Results: The overall malignancy rate was 1.63% (151/9269), increasing monotonically: 0.45% (under 50 years), 1.25% (50–59), 3.79% (60–69), and 4.58% (70 or older). Adjusted odds ratios versus the reference group were 2.02 (95% CI, 1.13–3.60), 5.77 (95% CI, 3.39–9.83), and 8.03 (95% CI, 4.51–14.30). Across all age groups, procedures with a documented postmenopausal bleeding (PMB) indication carried an overall malignancy rate of 6.32% (29/459; adjusted OR, 3.04; 95% CI, 1.96–4.72 versus incidental polyp finding); among women aged 70 or older with a documented PMB indication, the rate was 8/61 (13.1%; 95% CI, 6.8–23.8%). Procedures coded with a non-PMB uterine bleeding indication were not associated with excess malignancy risk (0.36%). Weight over 80 kg was an independent predictor of endometrial malignancy (adjusted OR, 3.05; 95% CI, 1.92–4.84). Age group and the documented PMB indication were entered as independent covariates in a single multivariable model; no significant age-by-PMB interaction was detected (likelihood ratio test *p* = 0.667). Conclusions: Malignancy risk rises more than tenfold from the youngest to the oldest age group. Procedures with a documented PMB indication were associated with substantially higher risk than other indications, while non-PMB bleeding was not associated with excess risk in this cohort. Age and documented procedural indication may be the most clinically useful predictors for stratifying histological examination decisions.

## Simple Summary

Among 9269 hysteroscopic polypectomies, endometrial malignancy rose from 0.45% in women under age 50 to 1.25% at ages 50–59, 3.79% at ages 60–69, and 4.58% at age 70 or older, reaching 8/61 (13.1%) in the oldest women with a documented postmenopausal bleeding (PMB) indication. Procedures coded with a non-PMB uterine bleeding indication were not associated with excess malignancy risk.

## 1. Introduction

The risk of endometrial malignancy in a polypectomy specimen has long been known to rise with age and postmenopausal status [1]. A 2025 systematic review and meta-analysis of 11,204 patients reported a malignancy rate of 2.75% (95% CI, 2.16–3.42) in peri- and postmenopausal women, identifying menopause (OR, 5.63; 95% CI, 3.87–8.20) and abnormal uterine bleeding (OR, 1.89; 95% CI, 1.06–3.35) as independent predictors [2]. A single-center retrospective study of 5839 patients similarly identified menopause (OR, 8.37), abnormal uterine bleeding (OR, 7.42), and obesity (OR, 3.22) as independent predictors [3]. A multi-institutional cohort of 813 patients found age per year (OR, 1.08) and abnormal uterine bleeding (OR, 3.53) to be independently significant [4]. Across these studies, advancing age and uterine bleeding emerge as the most consistent independent predictors of malignancy, yet absolute decade-specific estimates needed for pre-procedural counseling remain unavailable from any single cohort of comparable size.

These findings are consistent with a 2019 meta-analysis of 35,345 women reporting higher rates in postmenopausal than premenopausal women (4.93% vs. 1.12%) and in women with bleeding symptoms than without (5.14% vs. 1.89%) [5]. A meta-analysis of 21,057 patients identified age over 60 years (prevalence ratio, 2.41; 95% CI, 1.84–3.16), diabetes, and hypertension as independent risk factors [6]. Atypical hyperplasia confined to a completely resected polyp carries substantial additional risk: concurrent endometrial carcinoma was identified at hysterectomy in 30.8% to 50% of such cases [7,8]. Other series have examined clinical predictors of malignancy in endometrial polyps [9,10,11], but few have reported absolute rates by age decade.

Decade-specific absolute malignancy rates from large, unselected cohorts remain sparse, and few series have simultaneously examined how the procedural indication modifies the age-related gradient. Decade-specific absolute rates may help refine counseling across age groups, since current evidence suggests markedly different risk levels between, for example, a 72-year-old and a 54-year-old woman with a uterine polyp. Aggregate postmenopausal estimates may not fully reflect this gradient, which could be relevant to decisions about mandatory pathology submission, procedural urgency, or the threshold for additional endometrial sampling. Because the registry records the indication documented at the time of the procedure rather than menopausal status, the indication categories are interpreted throughout as documented indications. This analysis estimated the absolute malignancy rate for each age decade and compared malignancy rates between procedures with a documented postmenopausal bleeding (PMB) indication and those with a documented non-PMB abnormal uterine bleeding indication.

## 2. Materials and Methods

### 2.1. Study Design and Cohort

This was a retrospective cohort study with histology outcomes coded using the Systematized Nomenclature of Medicine (SNOMED). Data were extracted from the integrated electronic medical records of the Assuta Medical Network, a five-site private health system in Israel (Ramat HaHayal, Rishon LeZion, Ashdod, Beer Sheva, and Haifa). The observation period was January 2019 to April 2026. Procedures performed before January 2019 were excluded because SNOMED pathology linkage was absent in 2017 and only partially implemented in 2018 (18.6% linkage rate), which would have introduced systematic, non-age-related selection into the analytic cohort.

**Figure 1:**
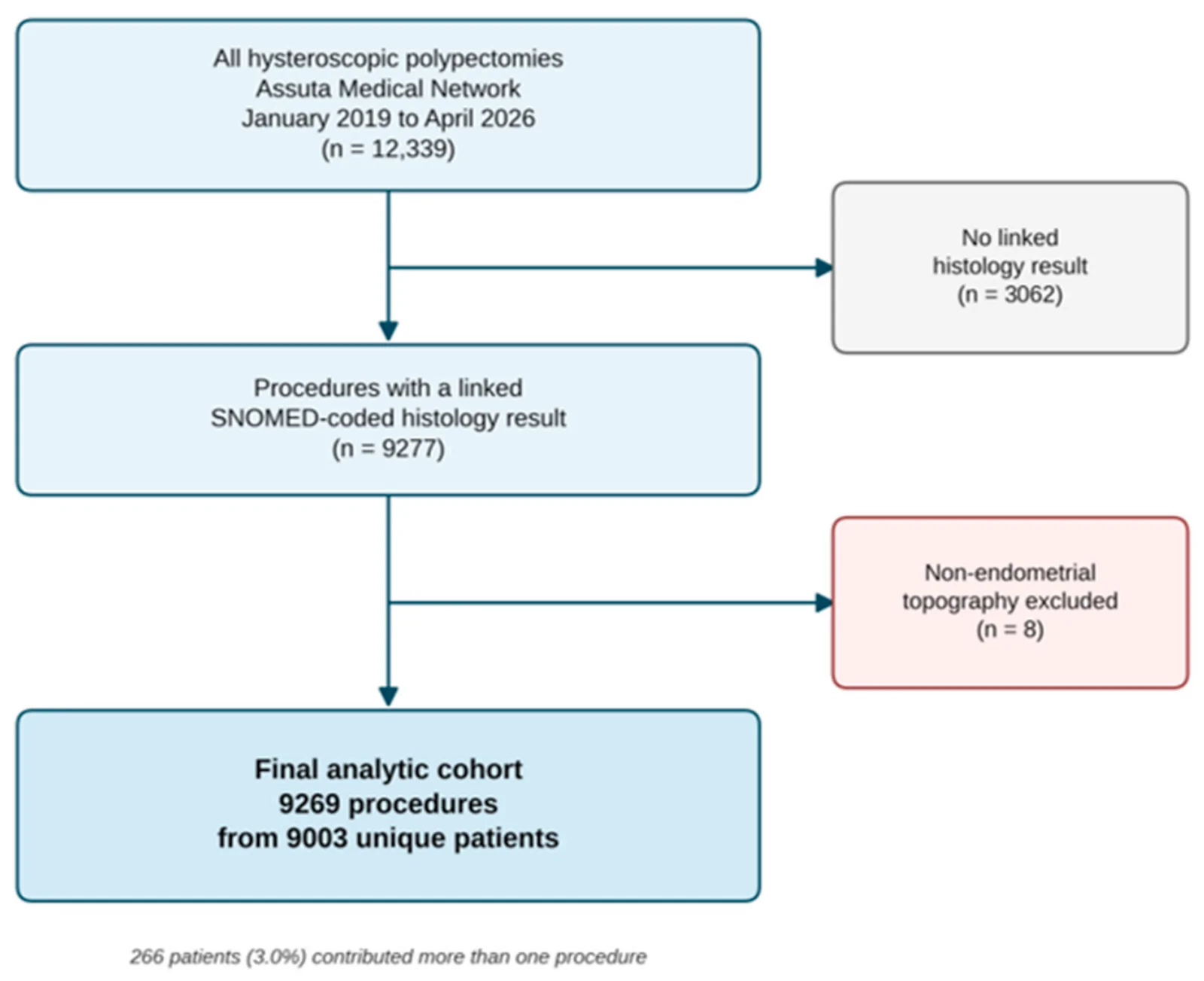
Flow of the study cohort from all hysteroscopic polypectomies in the Assuta Medical Network to the final analytic cohort.

The base cohort comprised all hysteroscopic polypectomies at any Assuta site during the study period (*n* = 12,339). Cases were identified by the operative procedure code assigned in the EMR at the time of the procedure; a pre-operative diagnosis of polyp by ultrasound was not required for inclusion. Among these, 9277 procedures (75.2%) had a linked SNOMED-coded histology result. Eight cases with non-endometrial topography (skin, breast, or urinary bladder) were identified in the morphology-topography linkage and excluded, yielding a final analytic cohort of 9269 procedures from 9003 unique patients (Figure 1). Procedures without a linked histology result (*n* = 3062) were not analyzed.

Histology submission rates, defined as the proportion of polypectomy procedures for which the excised specimen was sent for histological examination and yielded a linked SNOMED-coded result, were similar across age groups: 74.3% for women under 50, 76.1% for 50–59, 74.8% for 60–69, and 77.2% for 70 or older (chi-squared *p* = 0.23). Residual variation by calendar year (range, 63.6–91.1%) and hospital site (range, 57.2–80.7%), reflecting phased SNOMED implementation, was addressed by including both as covariates. Of 9003 unique patients, 266 (3.0%) contributed more than one polypectomy to the analytic cohort; the procedure was the unit of analysis. The statistical analysis plan was finalized before any outcome data were examined.

The polyp excision technique (mechanical scissors, bipolar electrosurgical instruments, or resectoscopic loop) was not standardized across sites and was selected at the discretion of the operating surgeon; procedures were performed by numerous gynecologic surgeons across the five sites, each applying the technique according to individual clinical judgment. The technical standard applied regardless of instrument was excision of the polyp including its base. Procedures were performed mainly by board-certified gynecologists with a minimum of three years of independent specialist experience; no trainees independently completed the procedure.

### 2.2. Outcome and Predictors

The primary outcome was histologically confirmed endometrial malignancy, defined as any SNOMED morphology record containing CARCINOMA, ADENOCARCINOMA, SARCOMA, or CARCINOSARCOMA, restricted to cases in which the corresponding topography field indicated endometrial or uterine body site. Eight cases with non-endometrial topography were excluded before defining the analytic cohort.

The primary predictor was patient age group at the time of the procedure: 18 to under 50 years (reference), 50–59, 60–69, and 70 or older. Age was analyzed in four categories rather than as a continuous variable, so that absolute rates could be reported by age group. Two clinical indication categories were secondary predictors: documented PMB indication and documented non-PMB abnormal uterine bleeding indication, each coded as binary from the primary EMR-documented procedural indication field. PMB reflects a documented procedural indication, not confirmed menopausal status; chronological age cannot be disentangled from time since menopause in this registry. Covariates were weight category (under 65 kg, 65–80 kg, over 80 kg, used as a body mass index surrogate because height data were unavailable), hospital site (five categories, fixed effect), and calendar year (continuous, centered at 2020).

### 2.3. Statistical Analysis

Logistic regression modeled malignancy risk as a function of age group, weight category, documented PMB status, hospital site, and calendar year. Age group and the documented PMB indication were entered simultaneously as independent covariates. A likelihood ratio test assessed whether the association of age with malignancy differed according to documented PMB status; no significant age-by-PMB interaction was detected (*p* = 0.667). Both unadjusted and adjusted odds ratios with 95% confidence intervals are reported. Absolute rates are presented with 95% Wilson score confidence intervals [12]. Model calibration was assessed with the Hosmer–Lemeshow goodness-of-fit test, which compares predicted with observed risks across risk strata.

With 148 events in the complete-case model and 11 estimated parameters, the events-per-variable ratio was 13.5, exceeding the recommended minimum of 10 [13]. Although this ratio supported the adequacy of the primary logistic model, an inverse probability weighting (IPW) sensitivity analysis was additionally performed to assess whether differential histology submission rates across age groups, calendar years, and hospital sites introduced selection bias. Selection weights were derived from a model of histology availability as a function of age group, calendar year, and site, truncated at the 99th percentile [14].

A sensitivity analysis restricted to each patient’s first polypectomy (*n* = 9003) assessed the potential influence of repeated procedures from the same patient. A Cochran–Armitage trend test evaluated the temporal stability of the annual unadjusted malignancy rate from 2019 to 2025 to assess whether the malignancy detection rate changed over time; 2026 was a partial year and was excluded from this test. The potential influence of unmeasured confounding on the age and PMB associations was quantified with E-values [15]. All analyses were performed in R (version 4.4). The threshold for statistical significance was *p* < 0.05 (two-tailed).

### 2.4. Ethical Approval

The study was conducted in accordance with the Declaration of Helsinki, and the protocol was approved by the Ethics Committee of Assuta Medical Centers (0042-26-ASMC) on 7 July 2026. Patient identifiers were replaced with study codes before data access. Individual informed consent was waived under Israeli regulations governing anonymized retrospective registry analyses.

## 3. Results

### 3.1. Cohort Characteristics

The analytic cohort comprised 9269 hysteroscopic polypectomies from 9003 unique patients (mean age, 52.1 years; SD, 11.7; range, 18–90). Women under 50 accounted for 4180 procedures (45.1%), followed by 2636 aged 50–59 (28.4%), 1689 aged 60–69 (18.2%), and 764 aged 70 or older (8.2%). Histology availability did not vary significantly by age group (*p* = 0.23). The annual unadjusted malignancy rate was stable across the study period (Cochran–Armitage trend test Z = −0.84, *p* = 0.40), and calendar year was not independently associated with malignancy in the adjusted model (OR, 0.985; 95% CI, 0.91–1.07; *p* = 0.72), suggesting that diagnostic improvements or case-mix shifts did not materially influence the observed rates.

Of the 9003 patients, 266 (3.0%) contributed more than one procedure; these procedures were distributed across calendar years without systematic clustering by indication or age. The sensitivity analysis restricted to each patient’s first procedure (*n* = 9003) produced adjusted odds ratios within 8% of those from the primary model across all age groups.

### 3.2. Age-Stratified Malignancy Rates

**Figure 2:**
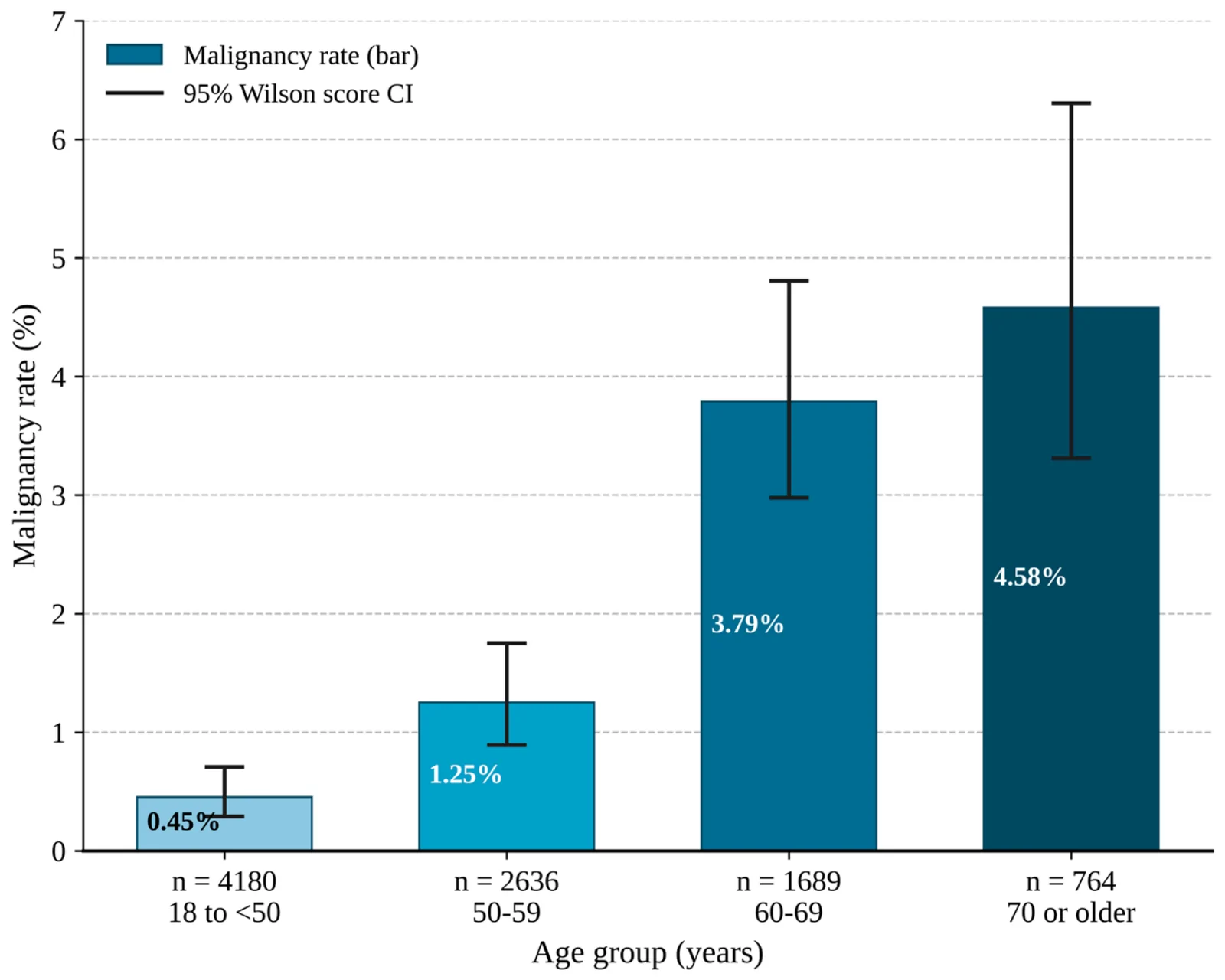
Absolute malignancy rate in hysteroscopic polypectomy specimens by age group, with 95% Wilson score confidence intervals. Rates were 0.45% (95% CI, 0.29–0.71%) for women aged 18 to under 50, 1.25% (95% CI, 0.89–1.75%) for 50–59, 3.79% (95% CI, 2.98–4.81%) for 60–69, and 4.58% (95% CI, 3.31–6.30%) for 70 or older. Group sizes are shown beneath each bar.

The overall malignancy rate was 1.63% (151/9269), with rates increasing monotonically across age decades (Table 1 and Figure 2). The adjusted odds ratio for the oldest group relative to the reference was 8.03 (95% CI, 4.51–14.30). Crude and adjusted odds ratios are shown separately in Table 1; the adjusted estimates were smaller than the crude estimates, which is consistent with confounding by weight and documented PMB status. Hyperplasia was recorded in 138 procedures (1.49%) overall, and hyperplasia rates did not increase significantly with age, ranging from 1.10% to 1.93% across age groups.

### 3.3. Malignancy Risk by Documented Procedural Indication

Malignancy rates varied by documented procedural indication (Table 2). Procedures with a documented PMB indication carried a rate of 6.32% (29/459; 95% CI, 4.39–9.00%; crude OR, 5.06; 95% CI, 3.26–7.87; adjusted OR, 3.04; 95% CI, 1.96–4.72) relative to incidental polyp finding. The adjusted odds ratio was lower than the crude estimate. Among women aged 70 or older with a documented PMB indication, the rate was 8/61 (13.1%; 95% CI, 6.8–23.8%), the highest in any subgroup. In each age group, the point estimate was higher with a documented PMB indication than without one, although the estimate for women under 50 rests on a single malignancy (Table 3). An age-by-PMB interaction test was not statistically significant (*p* = 0.667).

Procedures coded with a non-PMB abnormal uterine bleeding indication were not associated with excess malignancy risk (2/558; 0.36%; OR, 0.27; 95% CI, 0.07–1.10). Among the remaining indications, malignancy was found in 11 of 530 procedures performed for a suspicious ultrasound finding (2.08%), 2 of 80 for an intracavitary fibroid (2.50%), and 34 of 1849 with no coded indication (1.84%), compared with 73 of 5552 incidental polyp findings (1.31%). No malignancy was recorded among procedures coded for menorrhagia (*n* = 119), infertility workup (*n* = 84), or retained products of conception (*n* = 38). None of these categories differed significantly from incidental polyp findings.

The IPW sensitivity analysis yielded weighted odds ratios within 11% of the unweighted primary estimates across all age groups, and the direction and significance of all comparisons were preserved.

## 4. Discussion

Although postmenopausal status has long been recognized as a strong predictor of malignancy in endometrial polyps, with a reported odds ratio of 8.37 in a recent series [3], risk continued to increase well beyond the typical menopausal transition in this cohort. The adjusted odds ratio reached 8.03 for women aged 70 or older relative to those under 50, and the crude rate rose at each successive decade. The pooled postmenopausal malignancy rate from a 2019 meta-analysis of 35,345 women was 4.93% [5], similar to the rate in the oldest age group here (4.58%), but that estimate combines all postmenopausal women; in this cohort, rates ranged from 1.25% in the sixth decade to 4.58% in those aged 70 or older. Because the median age at natural menopause is approximately 51 years [16], the sixth decade spans the menopausal transition itself, meaning chronological age cannot fully substitute for confirmed menopausal status. Decade-specific estimates therefore describe a gradient that a single postmenopausal figure conceals, and this gradient may be relevant when women of different ages are counseled about the likelihood of an unexpected malignant finding.

Among women aged 70 or older with a documented PMB indication, the malignancy rate was 8/61 (13.1%; 95% CI, 6.8–23.8%), the highest in any subgroup. This exceeds the pooled estimate of approximately 3% for postmenopausal bleeding with polyps reported by Clarke et al. [17], and may reflect an age-specific amplification that pooled estimates do not capture. Malignancy risk in polyps has also been examined specifically in geriatric women [18]. The wide confidence interval and the non-significant age-by-PMB interaction indicate that this subgroup estimate should be regarded as descriptive. Procedures coded with a non-PMB bleeding indication were not associated with excess malignancy risk, a finding that refines the odds ratio of 7.42 reported by Jang et al. for abnormal uterine bleeding as a single undifferentiated category [3]. A systematic review restricted to premenopausal abnormal uterine bleeding reported comparably low absolute risk (intermenstrual bleeding: 0.52%; 95% CI, 0.23–1.16%) [19], consistent with these findings. Taken together, the data suggest that the excess risk attributed to abnormal uterine bleeding in pooled analyses may be concentrated among procedures with a documented PMB indication.

Weight over 80 kg (adjusted OR, 3.05) was associated with malignancy, consistent with published data [3,6,20,21]. In a prospective series of 421 women with postmenopausal bleeding and hysteroscopically benign-appearing polyps, Ghoubara et al. found body mass index and endometrial thickness to be the strongest predictors of malignancy or hyperplasia [22]. Whether obesity in this cohort acts partly through endometrial thickness cannot be determined from registry data, and weight category served only as a surrogate for body mass index. The association was retained after adjustment for age group, documented PMB status, site, and calendar year, but the categorical surrogate limits comparison with studies that used measured body mass index.

The malignancy rate rose more than tenfold from the youngest to the oldest age group, yet hyperplasia prevalence was essentially flat (1.10–1.93%). If these entities lay on a single continuum driven by cumulative estrogenic exposure, hyperplasia would be expected to track the malignancy rate. A possible explanation is that, in older women, a larger fraction of lesions that would earlier have been classified as hyperplasia had already progressed to carcinoma by the time of polypectomy; this explanation is speculative and cannot be tested without histologic grading. This registry did not distinguish hyperplasia with atypia from hyperplasia without atypia, a distinction with substantial prognostic weight [23], given that progression to carcinoma occurred in 23% of untreated atypical hyperplasia over 13 years versus 1.6% without atypia [24]. Population-based follow-up data likewise indicate a considerably higher risk of progression for atypical than for non-atypical hyperplasia [25].

The main findings were stable across the analyses performed to test their robustness. The annual malignancy rate did not change over the observation period, weighting for the probability of histology availability produced odds ratios within 11% of the primary estimates, and restriction to each patient’s first procedure produced odds ratios within 8% of the primary model. Histology submission rates were also similar across age groups, so differential submission by age is unlikely to account for the age gradient. These checks address selection and non-independence but cannot replace information on unmeasured clinical variables.

For clinical practice, these estimates may help clinicians approximate the pre-test likelihood of malignancy when counseling women before polyp removal, including decisions about pathology submission, procedural urgency, and the threshold for additional endometrial sampling. The data do not define thresholds for action, and they should be interpreted together with the individual clinical presentation. Strengths include a base cohort of all hysteroscopic polypectomies in a five-site network and an analysis plan finalized before outcome data were examined.

### Limitations

Menopausal status was not recorded; the documented PMB indication reflects an EMR-coded procedural entry, not a confirmed menopausal marker, and chronological age cannot be disentangled from time since menopause. SNOMED coding did not distinguish endometrial intraepithelial neoplasia from hyperplasia without atypia. The registry lacked data on nulliparity, polyp number and diameter, hormone therapy use, diabetes, hypertension, and endometrial thickness. Weight category was used as a body mass index surrogate because height was unavailable. Excision technique was not standardized across sites, and its possible influence on histological yield was not assessed. The age-by-PMB interaction was not significant; age-stratified PMB rates are presented as descriptive only.

About one quarter of the base cohort had no linked histology result. Weighting for age group, calendar year, and site did not change the main estimates, although selection related to unmeasured factors cannot be excluded. Of 9003 unique patients, 266 (3.0%) contributed more than one procedure; a sensitivity analysis restricted to the first procedure per patient yielded results consistent with the primary analysis, but this does not fully eliminate the possibility of within-patient correlation, and a clustered standard error approach could be considered in future work.

Residual confounding by unmeasured factors remains possible. For the age effect (adjusted OR, 8.03), the E-value was 15.5 (lower confidence bound, 8.5), and for the documented PMB indication (adjusted OR, 3.04) it was 5.5 (lower confidence bound, 3.3), with the odds ratios treated as approximations of risk ratios given the low outcome frequency. These values suggest that an unmeasured confounder would need to be associated with both the exposure and malignancy by a risk ratio of at least this magnitude to fully explain the observed associations. The cohort was also drawn from a single private health network in one country, and absolute rates may not be directly applicable to other settings.

## 5. Conclusions

Malignancy risk in polypectomy specimens rises steeply with age, corresponding to an adjusted odds ratio above 8 from women under 50 to those aged 70 or older. Among women aged 70 or older with a documented PMB procedural indication, the malignancy rate approached 13%. Procedures coded with a non-PMB uterine bleeding indication were not associated with excess malignancy risk in this cohort. Age and the documented PMB indication, rather than uterine bleeding as a single category, appeared to be the strongest clinical predictors of malignancy. These findings may support stratified decisions on histological examination in hysteroscopic polypectomy and help to judge the urgency of the procedure.

## Figures and Tables

**Table 1:** Age-stratified malignancy rates and hyperplasia frequency in hysteroscopic polypectomy specimens.

Age Group	Analytic *n* (%)	Malignancies, *n* (%; 95% CI)	Crude OR (95% CI)	Adjusted OR * (95% CI)	Hyperplasia, *n* (%)
18 to <50 (ref)	4180 (45.1%)	19 (0.45%; 0.29–0.71%)	1.00 (ref)	1.00 (ref)	46 (1.10%)
50–59	2636 (28.4%)	33 (1.25%; 0.89–1.75%)	2.78 (1.58–4.89)	2.02 (1.13–3.60)	51 (1.93%)
60–69	1689 (18.2%)	64 (3.79%; 2.98–4.81%)	8.63 (5.15–14.44)	5.77 (3.39–9.83)	32 (1.89%)
70 or older	764 (8.2%)	35 (4.58%; 3.31–6.30%)	10.51 (5.98–18.48)	8.03 (4.51–14.30)	9 (1.18%)
Total	9269	151 (1.63%)	--	--	138 (1.49%)

* Adjusted for weight category, hospital site, calendar year (centered 2020), and documented postmenopausal bleeding (PMB) indication. CI, confidence interval; OR, odds ratio; PMB, postmenopausal bleeding.

**Table 2:** Malignancy rates by documented procedural indication.

Indication	*n*	Malignancies, *n* (%)	Crude OR vs. Incidental (95% CI)	*p* Value
Postmenopausal bleeding	459	29 (6.32%)	5.06 (3.26–7.87)	<0.001
Suspicious ultrasound finding	530	11 (2.08%)	1.59 (0.84–3.02)	0.17
Intracavitary fibroid	80	2 (2.50%)	1.92 (0.46–7.98)	0.29
Incidental polyp (reference)	5552	73 (1.31%)	1.00 (reference)	--
Non-PMB abnormal uterine bleeding †	558	2 (0.36%)	0.27 (0.07–1.10)	0.07
Menorrhagia †	119	0 (0.00%)	Not calculable	>0.99
Infertility workup	84	0 (0.00%)	Not calculable	>0.99
Retained products of conception	38	0 (0.00%)	Not calculable	>0.99
No coded indication	1849	34 (1.84%)	1.41 (0.93–2.12)	0.11
Total	9269	151 (1.63%)	--	--

Fisher exact test was used for all comparisons. Postmenopausal bleeding adjusted OR (adjusted for age group, weight category, hospital site, and calendar year): 3.04 (95% CI, 1.96–4.72). PMB, postmenopausal bleeding; OR, odds ratio. † Non-PMB abnormal uterine bleeding and menorrhagia are mutually exclusive EMR-coded categories reflecting registry structure, not a clinical hierarchy.

**Table 3:** Age group by documented PMB status: procedures, malignancies, and absolute rates with 95% Wilson score confidence intervals.

Age Group	PMB: *n* (Procedures)	PMB: Malignancies *n* (%; 95% CI)	Non-PMB: *n* (Procedures)	Non-PMB: Malignancies *n* (%; 95% CI)
18 to <50	20	1 (5.0%; 0.9–23.6%)	4160	18 (0.43%; 0.27–0.68%)
50–59	238	7 (2.9%; 1.4–5.9%)	2398	26 (1.08%; 0.74–1.57%)
60–69	140	13 (9.3%; 5.5–15.2%)	1549	51 (3.29%; 2.52–4.27%)
70 or older	61	8 (13.1%; 6.8–23.8%)	703	27 (3.84%; 2.65–5.52%)
Total	459	29 (6.32%)	8810	122 (1.39%)

PMB, documented postmenopausal bleeding indication; CI, confidence interval. Non-PMB comprises all procedures without a documented PMB indication. Confidence intervals are 95% Wilson score intervals.

## Data Availability

The anonymized dataset is available from the corresponding author upon reasonable request, subject to institutional data-sharing agreement and Ethics Committee approval.
